# Lactation performance and feed utilization of Rahmani ewes fed with either a newly produced bacteriocin-like substance or a commercial bacteriocin

**DOI:** 10.1093/tas/txad010

**Published:** 2023-01-23

**Authors:** Hossam H Azzaz, Ahmed E Kholif, Ahmed M Abd El Tawab, Mohamed El-Sherbiny, Hussein A Murad, Noha A Hassaan, Einar Vargas-Bello-Pérez

**Affiliations:** Dairy Science Department, National Research Centre, Giza, Egypt; Dairy Science Department, National Research Centre, Giza, Egypt; Dairy Science Department, National Research Centre, Giza, Egypt; Dairy Science Department, National Research Centre, Giza, Egypt; Dairy Science Department, National Research Centre, Giza, Egypt; Department of Animal Production, National Research Centre, Giza, Egypt; School of Agriculture, Policy and Development New Agriculture Building, Berkshire, UK

**Keywords:** bacteriocin, feed utilization, milk production, nisin, ruminal fermentation

## Abstract

The aim of the present study was to compare the effect of feeding a newly produced bacteriocin**-**like substance from *Lactococcus lactis* ssp*. lactis* (PNP) with a commercial bacteriocin (NISEEN-S; CNP) in lactating Rahmani ewe diets. In experiment 1, the effects of four levels (500, 1,000, 1,500, and 2,000 unit/kg substrate, dry matter (DM) basis) of both bacteriocins on in vitro ruminal fermentation kinetics, total gas production (TGP), methane production (CH_4_), and nutrient degradability were determined. In experiment 2, 2 wk before the expected parturition, 30 multiparous lactating Rahmani ewes (mean ± SD: 2 ± 0.3 parity, 46.8 ± 2.5 kg body weight, 23 ± 2.7 mo of age, and 370 ± 13 g/d of previous milk production) were equally divided into three treatments in a complete randomized design for 90 d. The ewes in the control treatment were offered a diet composed of 600 g of concentrate feed mixture, 300 g berseem hay, and 100 g of faba bean straw (Control), or supplemented with produced bacteriocin like substance (PNP) or commercial (CNP) bacteriocin at 500 unit/kg feed (DM basis). In experiment 1, both PNP and CNP linearly and quadratically decreased (*P* < 0.001) CH_4_ production; however, PNP and CNP at 500 unit/kg feed quadratically increased fiber degradability (*P* < 0.01). In experiment 2, both PNP and CNP increased (*P* < 0.05) nutrient digestibility, and ruminal total volatile fatty acids, acetate, and propionate, while decreasing ruminal ammonia-N. The PNP treatment increased (*P* < 0.05) blood total proteins and albumin, while PNP and CNP treatments increase serum glucose. Both PNP and CNP treatments increased (*P* < 0.05) daily milk production and milk efficiency, without affecting the concentration of milk components. Both PNP and CNP are recommended to improve feed utilization and milk production, with superior results detected for PNP at 500 unit/kg feed daily.

## Teaser

This study showed that a newly produced bacteriocin-like substance from *Lactococcus lactis*ssp*. Lactis*improve ewes’ milk production. A newly produced bacteriocin-like substance from *Lactococcus lactis*ssp*. Lactis*reduce in vitro methane production.INTRODUCTION

In the dairy industry, many feed additives have been used to improve milk production and milk components, however, the safety of feed additives in milk and animal health is a critical issue for both dairy producers and consumers (Bampidis et al., 2022). Bacteriocin is a category of feed additives that has gained interest for animal nutritionists and microbiologists (Hernández-González et al., 2021). It has been considered “generally recognized as safe” for its use as a food preservative (Federal Register: 53 FR 11247, April 6, 1988) in human nutrition.

Bacteriocins are defined as small antimicrobial peptides ribosomal synthesized by Gram-positive and Gram-negative bacteria, that have great antimicrobial effects against a wide range of bacteria (Cotter et al., 2013; Hammami et al., 2013). The antimicrobial effects of bacteriocins are mainly on Gram-positive and some Gram-negative bacteria (Bennett et al., 2022). In the rumen, many bacterial species naturally produce bacteriocins (Russell and Mantovani, 2002); however, the majority have not been investigated yet. Feeding bacteriocins, as feed additives, boosts ruminal and intestinal bacteriocin concentrations and improves the efficacy of bacteriocin-producing bacteria in the gastrointestinal tract of animals and thus, they could be used as modifiers for rumen fermentation (Kobayashr et al., 2010), with almost no residues in milk after few hours of treatment (Wu et al., 2007).

The effect of adding bacteriocins to the diets of lactating animals is still unknown. In many experiments, bacteriocins have been used for the prevention of metabolic disorders such as lactic acidosis and bloat (Kobayashr et al., 2010), and the control of bacterial presence on the teat skin of dairy cows (Bennett et al., 2022). Shen et al. (2017) compared a common bacteriocin (i.e., nisin) with monensin on ruminal microbiota and observed that nisin greatly inhibited methane (CH_4_) production and decreased acetate/propionate ratio without affecting dry matter (DM) digestibility compared to monensin. Plasma concentrations of blood metabolites as indicators of nutritional status and energy and protein metabolism were evaluated by Shen et al. (2018) who reported nisin addition decreased uric acid without affecting other plasma metabolites.

For farmers, the high cost of bacteriocins production is an obstacle to their commercial use in dairy animals. The active bacteriocin-producing bacteria, production on the cheap substrate under the optimal conditions of growth medium may pave the way toward mass production and commercialization of bacteriocins as feed additives. Earlier, we have been successful in producing nisin economically on cheese-industry waste (permeate) using a local strain of *Lactococcus lactis* ssp. *Lactis* (under publication). We hypothesized that bacteriocin as a feed additive with antimicrobial activity will affect ruminal microbes, fermentation patterns, and nutrient digestion resulting in affecting the performance of lactating ewes. Therefore, the aim of the present study was to: 1) evaluate different levels of produced bacteriocin-like substance and commercial nisin (NISEEN-S) on in vitro fermentation, 2) *determine the effects of feeding the two bacteriocins (produced and NISEEN*-S) in lactating Rahmani ewes’ diets on feed intake, nutrient degradability, milk production and composition, and ruminal fermentation.

## MATERIALS AND METHODS

Management of the sheep was in accordance with the third edition (2010) of the guide of Agricultural Research and Teaching of Federation of Animal Science Societies, Champaign, IL, USA.

### Bacteriocin Production

*Lactococcus lactis* EGY_NRC4 (NCBI accession number MW856657 https://www.ncbi.nlm.nih.gov/nuccore/MW856657) was isolated from homemade cheeses for the production of bacteriocin on permeate (cheese industry waste) basal medium. The permeate basal medium contained 5.0 g yeast extract, 5.0 g peptone, 0.5 g magnesium sulfate, 3.0 g ammonium chloride, 2.5 g ascorbic acid, and 1.0 liter permeate. The initial pH of the production medium was adjusted to 5.7, sterilized at 110 °C for 10 min, then cooled and inoculated with *Lactococcus lactis* EGY_NRC4 at 5% inoculum size. After 48 h of culture incubation at 35 °C, the culture was centrifuged at 5,000 rpm for 15 min at 4 °C to obtain the culture supernatant which contained the produced bacteriocin-like substance.

The bioassay of the produced bacteriocin-like substance was conducted as described by Flôres et al. (2003). Briefly, one tube of *Lactococcus lactis* ssp. *cremoris* precultured was diluted with 300 mL of free basic medium with the following composition (w/v): 10 g sucrose, 10 g peptone, 10 g yeast extract, 10 g KH_2_PO_4_, 2 g NaCl, and 0.4 g Mg_2_SO_4_ 7H_2_O. A total of 5 mL of this culture was distributed in deep tubes containing 5 mL of the same basic free medium plus 1 mL of nisin solution containing different concentrations. In the control tube, the nisin solution was replaced by 1 mL of medium. All tubes were incubated at 37 °C without shaking for 6 h and their optical density was measured at 600 nm using spectrophotometer. The growth was stopped by injecting into each tube 1 mL of 0.004% of thiomersalate solution. A series of standards was set up in triplicate for each assay.

### Experiment 1 (In Vitro Fermentation)

Using a stomach tube, rumen liquor was obtained from three adult Rahmani sheep (51 ± 2.6 kg BW) fed a fixed amount of concentrate and berseem hay daily at 1:1 DM basis. The rumen contents (liquid and solid contents 1:1, v/v) were collected before morning feeding at 0700 h, kept in prewarmed thermo containers at 39 °C under anaerobic conditions. About 500 mL of ruminal fluid was collected from all ewes of each treatment. To avoid saliva contamination, the first 50 mL of the rumen fluid samples were discarded. The rumen fluid was mixed for 10 s, squeezed through four layers of cheese cloth, and maintained in a water bath at 39 °C under continuous carbon dioxide flushing until inoculation (Theodorou et al., 1994). Three incubation runs were performed in three different weeks. Rumen contents obtained from the three sheep were combined for each run.

A total mixed ration composed of (per kg DM) 600 g of concentrate feed mixture, 300 g berseem hay, and 100 g of faba bean straw was used as a substrate. The nutrient contents of feed ingredients and basal diet are shown in Table 1. The in vitro total gas production (TGP) assay was conducted as described by Theodorou et al. (1994) and adapted to the semi-automatic system of Mauricio et al. (1999). Ground substrate samples (500 mg of DM) were incubated in 120-mL serum bottles (six bottles per dose of additives at each time).

**Table 1.** Chemical composition of ingredients and diets (g/kg DM) fed to lactating ewes

	Concentrate feed mixture^a^	Berseem	Faba bean straw	Basal diet^b^
Dry matter	883	968	878	908
Organic matter	941	844	905	908
Crude protein	143	95	69	121
Ether extract	57	29	33	46
Nonstructural carbohydrates	451	253	190	365
Neutral detergent fiber	291	467	613	376
Acid detergent fiber	100	308	450	197

^a^Contained per kg DM: The concentrate feed mixture consisted of (per kg DM): 350 g corn, 440 g wheat bran, 180 g soybean meal, 20 g limestone, 5 g sodium chloride, and 5 g minerals and vitamins mixture [containing per kg: 141 g Ca, 87 g P, 45 g Mg, 14 g S, 120 g Na, 6 g K, 944 mg Fe, 1613 mg Zn, 484 mg Cu, 1748 mg Mn, 58 mg I, 51 mg Co, 13 mg Se, 248,000 IU vitamin A, 74,000 IU vitamin D3, 1656 IU vitamin E].

^b^Diet: Control diet contained 600 g of concentrate feed mixture, 300 g berseem hay, and 100 g of faba bean straw.

The incubated substrate without additives was considered the control treatment. The bacteriocin-like substance produced from *Lactococcus lactis* (PNP) and the commercial bacteriocin (NISEEN-S product of Siveele B.V, the Netherlands) were evaluated at 500, 1,000, 1,500, or 2,000 unit/kg substrates (DM basis). Levels of inclusion were based on the initial screening of many doses of the additives. After dispensing, bottles were closed with rubber stoppers, shaken manually, and incubated at 39 °C in a forced-air oven for 48 h. The bottles were shaken at 1 h intervals during incubation. The amount of GP was calculated according to the regression equation (*V* = 4.974 12 × *p* + 0.171; *n* = 500, *r*^2^ = 0.98; [V is the gas volume (mL); *p* is measured pressure (psi)]) obtained in our laboratory under our conditions according to the gas volume vs. pressure. Bottles containing only buffered rumen fluid without substrate were considered blanks. At each incubation time, 5 mL of headspace gas was taken from each bottle and infused into a Gas-Pro detector (Gas Analyzer CROWCON Model Tetra3, Abingdon, UK) to measure the concentration of CH_4_. The control and experimental treatments were tested in six bottles (analytical replicates) and three incubation runs in three consecutive weeks with three bottles containing inoculum and buffer but no feed (blanks).

At 48 h (the end of incubation), fermentation was terminated by immersing the bottles in ice. For each additives level, three bottles were used to measure the pH, ammonia-N (AOAC, 2005), and volatile fatty acids (VFA) by steam distillation and titration (Warner, 1964), whereas the other three bottles were filtered in preweighed crucibles and washed with hot water then acetone, and the residual DM and ash were estimated to determine true DM, organic matter (OM), neutral detergent fiber (NDF), and acid detergent fiber (ADF) degradability (dDM, dOM, dNDF, and dADF, respectively).

### Experiment 2 (Animal Performance)

#### Ewes and management

Two weeks before expected parturition, 30 multiparous lactating Rahmani ewes (mean ± SD: 2 ± 0.3 parity, 46.8 ± 2.5 kg body weight, 23 ± 2.7 months of age, and 370 ± 13 g/d of previous milk production) were assigned randomly to three dietary treatments (*n* = 10 ewes/treatment). Ewes were randomly allocated to treatments in a completely randomized design. Ewes were individually kept in semi-opened concrete floor pens (1.5 m^2^/sheep) with free access to water. Sheep were fed a basal diet (Control) comprising of concentrate feed mixture, berseem hay, and faba bean straw at 60:30:10, respectively (DM basis) without additives. For the rest of the treatments, ewes were fed the control diet supplemented with the bacteriocin-like substance produced by *Lactococcus lactis* (PNP treatment) or commercial (NISEEN-S product of Siveele B.V, the Netherlands) bacteriocin (CNP treatment) at 500 U/kg feed (DM basis).

Diets were offered to the animals individually at 08:00 and 16:00 h in two equal amounts. Ewes were first offered the allotted amounts of concentrate feed mixture, followed by berseem hay and faba bean straw after the consumption of concentrate feed. Diets were prepared to meet nutrient requirements for milk production of ewes according to NRC (2007) recommendations. To ensure orts collection, feeds were offered 1.10 times above the NRC (2007) recommendations. The experiment lasted for 90 d. Individual animals were weighed at monthly intervals. Table 1 shows the ingredient and chemical compositions of the experimental diets. The daily samples of diets were composited weekly and dried at 60 °C in a forced-air oven for 48 h (AOAC, 2005) (method 930.15) before storing for chemical analyses.

#### Feed intake and nutrient apparent digestibility

Three digestibility trials were conducted during the last 10 d of each month using acid-insoluble ash as an internal indigestibility marker. The equations of Ferret et al. (1999) were used to calculate the coefficients of apparent digestion. Feed intake was calculated as the difference between feed offered and orts from the previous day’s feeding. Individual fecal grab samples were collected twice daily during the collection period at 07:00 and 15:00 h, dried at 60 °C in a forced-air oven for 48 h and pooled per ewe.

Composited samples of dried feeds, orts, and feces were ground to pass through a 1-mm screen using a mill and analyzed for DM, ash, nitrogen, and ether extract (EE) according to AOAC (2005) official methods. NDF content was determined according to Van Soest et al. (1991). ADF content was analyzed according to AOAC (2005) and expressed exclusive residual ash. Nonstructural carbohydrates (NSC) and OM concentrations were calculated. Diet’s nutritive value was calculated according to the equations of NRC (2001) as: total digestible nutrients (TDN), digestible energy (DE), metabolizable energy (ME), and net energy for lactation (NEL). The net energy requirements for lactation equivalent to 1 kg of standard air-dried barley (UFL; *unité fourragère du lait*) was calculated according to INRA (2018) equation.

#### Sampling and analysis of rumen fluid

On days 30, 60, and 90 of the experiment, ruminal fluid samples were collected from all animals in the morning at 3 h postfeeding (at 11:00 h) to analyze the concentrations of VFA and ammonia-N. About 100 mL of ruminal fluid was collected with stomach tube from each ewe, and the first 50 mL of the rumen fluid samples were discarded to avoid saliva contamination, and the rumen contents were strained through four layers of cheesecloth. The rumen fluid pH was measured immediately using a pH meter (HI98127 pHep 4 pH/Temperature Tester, Hanna Instruments, Villafranca padovana PD, Italy). Five mL of subsample were preserved in 5 mL of 0.2 mol HCl for ammonia-N analysis and 0.8 mL of rumen fluid was mixed with 0.2 mL of a solution containing 250 g of metaphosphoric acid/L for total VFA analysis. The collected samples were preserved at –20 ºC pending analyses. The rumen liquor was used for ammonia-N analysis according to the AOAC (2005). Concentration of VFA and its individual molar proportions were determined using a gas chromatograph (Thermo fisher scientific, Inc., TRACE1300, Rodano, Milan, Italy) fitted with an AS3800 autosampler and equipped with a capillary column HP-FFAP (19091F-112; 0.320 mm o.d., 0.50 μm i.d., and 25 m length; J & W Agilent Technologies Inc., Palo Alto, CA, USA). A mixture of known concentrations of individual short-chain fatty acid VFAs was used as an external standard (Sigma Chemie GmbH, Steinheim, Germany) to calibrate the integrator.

#### Sampling and analysis of blood serum

On days 30, 60, and 90 of the experiment, blood samples (10 mL) were collected at 4 h postfeeding (at 12:00 h) from the jugular vein of each ewe into clean dry tubes without anticoagulants. Collected samples were centrifuged at 4,000 × *g* for 20 min at 4 ºC, and serum was decanted into 2-mL Eppendorf tubes and frozen at –20 ºC pending analysis using specific kits (Stanbio Laboratory, Boerne, Texas, USA) according to manufacturer instructions. Samples were analyzed for total proteins, albumin, urea-N, glucose, cholesterol, glutamatepyruvate transaminase (GPT), and glutamate-oxaloacetate transaminase (GOT). Globulin concentration was calculated (total protein—albumin).

#### Milk sampling and composition

Ewes were hand-milked individually during the last 10 d of each experimental period at 09:00 and 21:00 h, and 10% of recorded milk yield samples were taken at each milking. A mixed sample of morning and evening milkings was taken daily, composited for the immediate analysis of milk components (fat, lactose, total solids, and protein) using infrared spectrophotometry (Lactostar Dairy Analyzer, Funke Gerber, Berlin, Germany).

Gross energy content in milk, fat-corrected milk (FCM, kg/day), and energy-corrected milk (ECM, kg/day) was calculated according to Tyrrell and Reid (1965). Feed efficiency was calculated and expressed as milk yield, FCM and ECM per unit of DM intake.

### Statistical Analyses

The Shapiro–Wilk test was used to test the normal distribution of data. For the small number of variables that showed significance for the Shapiro–Wilk test (serum globulin and cholesterol), data transformation (natural log) was applied before statistical analysis.

Data from in vitro measurements were analyzed using the GLM procedure of SAS (SAS Inst. Inc. Cary, NC, USA) in a completely randomized design using the following model: *Y*_*ij*_ = μ + *D*_*i*_ + *e*_*ij*_, where *Y*_*ij*_ represents the measured variable, μ is the overall mean, *D*_*i*_ is the additive dose, and *e*_*ij*_ is the experimental error. Data from each of the three runs within the same sample were averaged prior to the statistical analysis. Polynomial (linear and quadratic) contrasts were used to examine dose responses for increasing levels of additives.

Data of the lactation experiment were analyzed using a completely randomized design with repeated measurements in time, where each ewe was an experimental unit using PROC MIXED of SAS (Online Version, SAS OnDemand for Academics, SAS Inst., Inc., Cary, NC). The following model was used as:


$$\begin{array}{l} Y_{ijkl}=\mu +\;T_{i}+\;A_{j}\left(T_{i}\right)\;+\;P_{k}+\;{\left(T\times P\right)}_{ik}+\;e_{ijkl}, \end{array}$$

where *Y*_*ijkl*_ expressed each observation of the *j*th ewe in the *k*th sampling time given *i*th diet, *T*_*i*_ expressed the effect of diets, *A*(*T*)_*ji*_ expressed the ewe within each diet, *P*_*k*_ expressed the sampling week effect, (*T* × *P*)_ik_ expressed the interaction between the diets and sampling period, and *e*_*ijkl*_ expressed the experimental error. Additionally, the contrast between produced-like substance and commercial bacteriocins were applied. The diet × period interactions were nonsignificant (i.e., *P* > 0.05) for most of the measurements; thus, only the main effects of diets and periods were reported. Significance was declared at a level of *P* < 0.05.

## RESULTS

### Experiment 1 (In Vitro Fermentation)

Compared to the control diet, PNP at 500, 1,000, and 1,500 unit/kg DM of feed (linear and quadratic effects, *P* < 0.01) and CNP at 500 unit/kg DM of feed (quadratic effect, *P* < 0.001) increased TGP (Table 2). Compared to the control, all inclusion levels of PNP and CNP linearly and quadratically decreased CH_4_ production. The lowest level (500 unit/g DM feed) of PNP and CNP (*P* < 0.01) quadratically increased dNDF and dADF; however, PNP at 500 unit/g DM feed also increased dDM and dOM, with no effects observed with the other levels of PNP and CNP. PNP and CNP did not affect fermentation pH; however, PNP at 500, 1,000, and 1,500 unit/kg DM of feed (linear and quadratic effects, *P* < 0.001) and CNP at 500 unit/kg DM of feed (quadratic effect, *P* < 0.001) increased VFA concentration, and all levels of PNP and CNP linearly and quadratically decreased (*P* < 0.05) NH_3_–N concentrations.

**Table 2.** In vitro fermentation *parameters of the control diet supplemented with bacteriocin like substance* produced by *Lactococcus lactis* (PNP treatment) or from the commercial product (CNP treatment) at 500, 1,000, 1,500, or 2,000 unit/kg feed (DM basis)

		PNP				CNP				SEM	PNP		CNP		PNP vs. CNP
	Control	500	1,000	1,500	2,000	500	1,000	1,500	2,000		Linear	Quadratic	Linear	Quadratic	
TGP, mL/g DM	106^c^	118^a^	113^b^	113^b^	102^c^	119^a^	110^c^	108^c^	103^c^	0.9	0.001	<0.001	0.815	<0.001	0.016
CH_4_, %	24.6^a^	16.7^b^	16.3^b^	15.7^b^	14.5^b^	16.2^b^	17.5^b^	17.0^b^	16.6^b^	0.50	<0.001	<0.001	<0.001	<0.001	0.008
CH_4_/g dDM	556^a^	394^b^	383^b^	379^b^	333^b^	393^b^	403^b^	390^b^	379^b^	11.6	<0.001	<0.001	<0.001	<0.001	0.022
CH_4_/g dOM	471^a^	339^b^	328^b^	319^b^	278^b^	338^b^	341^b^	325^b^	322^b^	9.9	<0.001	<0.001	<0.001	<0.001	0.036
CH_4_/g dNDF	712^a^	494^b^	485^b^	484^b^	431^b^	497^b^	513^b^	498^b^	490^b^	14.8	<0.001	<0.001	<0.001	<0.001	0.018
CH_4_/g dADF	937^a^	633^b^	629^b^	636^b^	577^b^	642^b^	668^b^	654^b^	652^b^	20.4	<0.001	<0.001	<0.001	<0.001	0.019
dDM, g/kg	46.6^b^	50.3^a^	48.4^b^	46.8^b^	44.7^b^	49.0^b^	47.9^b^	46.9^b^	45.3^b^	0.32	0.142	<0.001	0.137	0.155	0.196
dOM, g/kg	55.0^b^	58.4^a^	56.5^b^	55.6^b^	53.5^b^	57.1^b^	56.6^b^	56.3^b^	53.4^b^	0.35	0.133	<0.001	0.314	0.236	0.608
dNDF, g/kg	36.4^b^	40.1^a^	38.2^b^	36.6^b^	34.5^b^	38.8^a^	37.6^b^	36.7^b^	35.1^b^	0.32	0.551	<0.001	0.111	0.001	0.196
dADF, g/kg	27.7^b^	31.3^a^	29.5^b^	27.9^b^	25.8^b^	30.1^a^	28.9^b^	28.0^b^	26.4^b^	0.32	0.071	<0.001	0.160	0.001	0.196
pH	6.60	6.40	6.51	6.59	6.65	6.47	6.51	6.57	6.63	0.017	0.881	0.824	0.121	0.350	0.438
VFA, mmol/L	117^b^	137^a^	128^a^	127^a^	109^c^	129^a^	122^b^	118^b^	113^b^	0.92	<0.001	<0.001	0.222	<0.001	<0.001
NH_3_-N, mg/dL	13.26^a^	11.84^b^	11.74^b^	11.49^b^	11.12^b^	11.77^b^	11.65^b^	11.42^b^	11.08^b^	0.174b	<0.001	0.012	0.004	<0.001	0.609

Means in the same row with different superscripts differ, *P* < 0.05. *P*-value is the observed significance level of the *F*-test for treatment; SEM, standard error of the mean.

dAFD, acid detergent fiber degradability; CH_4_, methane; dDM, dry matter degradability; dNDF, neutral detergent fiber degradability; dOM, organic matter degradability (g/kg); VFA, volatile fatty acids (mmol/L); TGP, total gas production (mL/g DM).

### Experiment 2 (Animal Performance)

#### Feed intake and apparent nutrient digestibility

PNP and CNP did not affect feed intake (Table 3). PNP followed by CNP increased DM, OM, and NDF digestibility. Compared to the control, both PNP and CNP increased NSC and ADF digestibility. PNP followed by CNP increased the nutritive value of diets calculated as TDN, DE, ME, NEL, and UFL.

**Table 3.** Intake and nutrient digestibility of diets supplemented with bacteriocin like substance produced from *Lactococcus lactis* (PNP treatment) or from the commercial product (CNP treatment) at 500 unit/kg feed (DM basis) in lactating Rahmani ewes

	Diet^a^			SEM	*P* value			
	Control	PNP	CNP		Diet	Period	Control vs. others	PNP vs. CNP
Intake, g/ewe/d								
Concentrates	744	741	731	16.8	0.684	0.653	0.831	0.000
Berseem hay	374	377	369	8.2	0.816	<0.0001	0.904	0.533
Bean straw	125	127	127	3.0	0.822	0.006	0.538	0.922
Total	1243	1245	1227	24.5	0.854	<0.0001	0.808	0.614
Digestibility, g absorbed/kg ingested								
Dry matter	628^c^	698^a^	660^b^	8.5	<0.001	0.990	<0.001	0.003
Organic matter	635^c^	726^a^	689^b^	7.5	<0.001	0.685	<0.001	0.001
Crude protein	629	670	647	14.1	0.131	0.967	0.097	0.248
Ether extract	664	676	680	13.4	0.710	0.008	0.423	0.845
Nonstructural carbohydrates	591^b^	655^a^	629^a^	10.7	0.004	0.292	0.003	0.095
Neutral detergent fiber	587^c^	673^a^	641^b^	9.3	<0.001	0.687	<0.001	0.021
Acid detergent fiber	595^b^	666^a^	649^a^	11.5	0.001	0.335	<0.001	0.277
Nutritive value								
TDN, g/kg DM^b^	581^c^	646^a^	622^b^	8.5	<0.001	0.669	<0.001	0.045
DE, Mcal/kg DM^b^	2.56^c^	2.85^a^	2.74^b^	0.037	<0.001	0.650	<0.001	0.045
ME, Mcal/kg DM^b^	2.59^c^	2.88^a^	2.77^b^	0.038	<0.001	0.692	<0.001	0.041
NEL, Mcal/kg DM^b^	1.30^c^	1.46^a^	1.40^b^	0.021	<0.001	0.648	<0.001	0.039
UFL, Mcal/kg DM^c^	2.30^c^	2.58^a^	2.47^b^	0.036	<0.001	0.676	<0.001	0.044

Means in the same row with different superscripts differ (*P* < 0.05).

SEM, standard error of the mean.

^a^Diet: Control diet contained 600 g of concentrate feed mixture, 300 g berseem hay and 100 g of faba bean straw (Control), or supplemented with bacteriocin like substance produced by *Lactococcus lactis* (PNP treatment) or from the commercial product (CNP treatment) at 500 unit/kg feed (DM basis).

^b^ TDN, total digestible nutrients: DE, digestible energy; ME, metabolizable energy; NEL, net energy for lactation. All have been calculated according to NRC (2001) equation.

^c^ UFL, *unité fourragère du lait* (net energy requirements for lactation equivalent of 1 kg of standard air-dried barley) calculated according to INRA (2018) equation.

#### Ruminal fermentation

PNP and CNP did not affect ruminal pH (Table 4). Both PNP and CNP lowered ruminal NH_3_–N (*P* < 0.001); however, they increased VFA (*P* < 0.001), acetate (*P* < 0.001), and propionate (*P* = 0.01) without affecting butyrate or acetate: propionate ratio.

**Table 4.** Ruminal fermentation of lactating Rahmani ewes fed diet supplemented with bacteriocin like substance produced by *Lactococcus lactis* (PNP treatment) or from the commercial product (CNP treatment) at 500 unit/kg feed (DM basis)

	Diet^a^			SEM	*P* value			
	Control	PNP	CNP		Diet	Period	Control vs. others	PNP vs. CNP
pH	6.13	6.07	6.09	0.039	0.514	0.766	0.283	0.682
Ammonia-N, mg/dL	27.7^a^	25.2^b^	25.7^b^	0.34	<0.001	0.314	<0.001	0.288
Total Short chain fatty acids, mmol/L	105^b^	114^a^	114^a^	1.2	<0.001	0.463	<0.001	0.976
Acetate, mmol/L	63.1^b^	70.2^a^	69.5^a^	0.97	<0.001	0.931	<0.001	0.640
Propionate, mmol/L	29.9^b^	32.6^a^	32.7^a^	0.72	0.010	0.367	0.003	0.904
Butyrate, mmol/L	11.8	11.5	12.0	0.68	0.887	0.039	0.965	0.627
Acetate: propionate ratio	2.14	2.17	2.14	0.057	0.897	0.343	0.792	0.702

Means in the same row with different superscripts differ (*P* < 0.05).

^a^Diet: Control diet contained 600 g of concentrate feed mixture, 300 g berseem hay and 100 g of faba bean straw (Control), or supplemented with bacteriocin like substance produced by *Lactococcus lactis* (PNP treatment) or from the commercial product (CNP treatment) at 500 unit/kg of feed (DM basis).

#### Blood chemistry

PNP treatment increased blood total proteins (*P* < 0.001) and albumin (*P* = 0.001) compared to control and CNP treatments (Table 5). Both PNP and CNP treatments increased serum globulin (*P* = 0.021) and glucose (*P* < 0.001). Treatments did not affect concentrations of serum albumin: globulin ratio, urea-N, cholesterol, GOT, and GPT.

**Table 5.** Blood measurements (mg/dL, unless stated otherwise) of lactating Rahmani ewes fed diet supplemented with bacteriocin like substance produced by *Lactococcus lactis* (PNP treatment) or from the commercial product (CNP treatment) at 500 unit/kg feed (DM basis)

	Diet^a^			SEM	*P*-value			
	Control	PNP	CNP		Diet	Period	Control vs. others	PNP vs. CNP
Total proteins	6.79^b^	7.40^a^	7.04^b^	0.081	<0.001	0.005	<0.001	0.003
Albumin	3.79^b^	4.12^a^	3.89^b^	0.061	0.001	0.151	0.006	0.012
Globulin	3.00^b^	3.28^a^	3.15^a^	0.069	0.021	0.033	0.014	0.180
Albumin: globulin ratio	1.27	1.27	1.25	0.037	0.933	0.489	0.894	0.729
Urea-N	48.3	47.0	46.2	1.33	0.521	0.201	0.289	0.678
Glucose	73.9^b^	81.3^a^	78.4^a^	1.12	<0.001	0.039	<0.001	0.079
Cholesterol	99.5	106.4	104.7	4.98	0.597	0.054	0.327	0.804
GOT, Units/L	36.7	37.9	37.4	1.56	0.853	0.221	0.606	0.823
GPT, Units/L	19.9	21.2	21.0	0.64	0.288	0.813	0.121	0.800

GOT, glutamate-oxaloacetate transaminase; GPT, glutamate-pyruvate transaminase.

Means in the same row with different superscripts differ (*P* < 0.05).

^a^Diet: Control diet contained 600 g of concentrate feed mixture, 300 g berseem hay and 100 g of faba bean straw (Control), or supplemented with bacteriocin produced by *Lactococcus lactis* (PNP treatment) or from the commercial product (CNP treatment) at 500 unit/kg feed (DM basis).

#### Milk yield and composition

Both PNP and CNP treatments increased (*P* < 0.05) daily production of milk, ECM, FCM, and energy output and yields all milk components except for fat (Table 6). Both treatments improved feed efficiency calculated as milk: intake, ECM: intake, and FCM: intake.

**Table 6.** Milk production, composition, and feed efficiency of lactating Rahmani ewes fed diet supplemented with bacteriocin like substance produced by *Lactococcus lactis* (PNP treatment) or from the commercial product (CNP treatment) at 500 unit/kg feed (DM basis)

	Diet^a^			SEM	*P* value			
	Control	PNP	CNP		Diet	Period	Control vs. others	PNP vs. CNP
Production, g/d								
Milk	360^b^	398^a^	390^a^	10.9	0.026	<0.001	0.010	0.672
Energy corrected milk, ECM	423^b^	471^a^	454^a^	8.1	0.033	<0.001	0.015	0.506
Fat corrected milk 4%, FCM	391^b^	444^a^	424^a^	8.4	0.024	<0.001	0.011	0.448
Total solids	55.9^b^	61.6^a^	59.9^a^	0.21	0.036	<0.001	0.016	0.578
Solids nonfat	39.5^b^	42.6^a^	42.0^a^	0.42	0.022	<0.001	0.022	0.747
Protein	13.5	14.4	14.2	0.48	0.068	<0.001	0.055	0.749
Fat	16.4^b^	19.0^a^	17.9^a^	0.91	0.029	0.001	0.015	0.397
Lactose	22.0^b^	23.9^a^	23.5^a^	0.40	0.046	<0.001	0.019	0.738
Milk energy output, MJ/d	1.33^b^	1.48^a^	1.43^a^	0.037	0.034	<0.001	0.016	0.505
Composition, g/kg DM								
Total solids	155	155	153	1.6	0.629	0.014	0.623	0.408
Solids nonfat	110^a^	108^b^	108^b^	0.6	0.043	0.005	0.013	0.831
Protein	37.4^a^	36.4^b^	36.4^b^	0.26	0.012	0.024	0.003	0.881
Fat	45.4	47.5	45.4	1.41	0.479	0.164	0.552	0.291
Lactose	61.2	60.2	60.3	0.36	0.123	0.001	0.042	0.914
Milk energy content, MJ/kg DM	3.69	3.73	3.65	0.058	0.614	0.042	1.000	0.324
Feed efficiency								
Milk: intake	0.29^b^	0.32^a^	0.32^a^	0.001	<0.001	0.035	<0.001	0.932
ECM: intake	0.34^b^	0.38^a^	0.37^a^	0.001	<0.001	0.023	<0.001	0.888
FCM: intake	0.31^b^	0.36^a^	0.35^a^	0.001	<0.001	0.033	<0.001	0.903

Means in the same row with different superscripts differ (*P* < 0.05).

^a^Diet: Control diet contained 600 g of concentrate feed mixture, 300 g berseem hay and 100 g of faba bean straw (Control), or supplemented with bacteriocin like substance produced by *Lactococcus lactis* (PNP treatment) or from the commercial product (CNP treatment) at 500 unit/kg feed (DM basis).

## DISCUSSION

### Experiment 1 (In Vitro Fermentation)

Unexpectedly and without a clear reason, PNP (except for the 2,000 unit/g DM feed level), and CNP at 500 unit/g DM feed increased TGP indicating improved rumen emphasizing the importance of defining the optimal dose of each bacteriocin. Shen et al., (2017) observed lowered TGP with in vitro nisin administration at 5 μM from a nisin product containing 1,200 IU/mg of incubated feed. However, all levels of inclusion of PNP and CNP linearly decreased CH_4_ production indicating a high antimicrobial activity against methanogens (Shen et al., 2017). Lee et al. (2002a) and Shen et al. (2017) stated that the inclusion of bacteriocins decreased CH_4_ production by more than 50% because of the reduced population of methanogens. Bacteriocins can lower the numbers of many H_2_-producing microorganisms (e.g., some protozoa, fungi, and Gram-positive taxa of Firmicutes species), which reduces the availability of H_2_ for CH_4_ production (Shen et al., 2017). Moreover, the ability of bacteriocins to alter ruminal fermentation toward increasing propionate production (Hernández-González et al., 2021) may be considered as another reason for the lowered CH_4_ production (Choudhury et al., 2022) observed in the present study. Increasing gas production and improving nutrient degradability while lowering CH_4_ production is solid evidence from the selectivity of bacteriocins against ruminal microbes (Lee et al., 2002a; Kobayashr et al., 2010).

PNP and CNP at 500 unit/kg feed increased dNDF and dADF, dDM, and dOM, which may be related to changes in the rumen microbial composition of animals fed bacteriocins ( Lee et al., 2002b; Shen et al., 2017). The selective antimicrobial activity of bacteriocins against bacteria (Kierończyk et al., 2020) may be the reason for lowering some species without affecting other species that may be responsible for nutrient digestion (Hernández-González et al., 2021). A relative abundance of *Pseudobutyrivibrio*, *Butyrivibrio*, *Rikenellaceae*, *Bacteroidales* (Shen et al., 2017), *Megasphaera elsdenii*, *Fibrobacter succinogenes*, *Ruminococcus albus*, *Ehrlichia ruminantium*, and *Streptococcus bovis* (Demirtaş, 2020) which have vital roles in nutrient digestion were observed with nisin feeding to animals.

Both evaluated bacteriocins at all levels decreased NH_3_–N concentrations, which may be related to the ability of bacteriocins to suppress amino acid deamination which may limit its utilization (Russell and Mantovani, 2002). At the same time, PNP at 500, 1,000, and 1,500 unit/kg DM of feed and CNP at 500 unit/kg DM of feed increased VFA concentration, which is paralleled with the observed results on nutrient degradability. Improving nutrient degradability increases VFA production (France and Dijkstra, 2005). Shen et al. (2017) stated that nisin affects the composition of ruminal microbes which increases the production of some VFA resulting in increased production of total VFA.

In the present in vitro experiment, the effects of PNP were linear, with almost no differences between doses; therefore, the lowest dose of PNP was chosen for the lactation experiment. Additionally, the lowest dose of CNP quadratically affected the measured fermentation parameters; therefore, this level of inclusion was used in the lactation performance.

### Experiment 2 (Lactation Performance)

#### Feed intake and nutrient apparent digestibility

PNP and CNP did not affect feed intake indicating unaffected animal’s acceptability with bacteriocins; however, PNP followed by CNP increased nutrient digestibility and nutritive value because of changing the microbial composition in ewes fed bacteriocins (Lee et al., 2002b; Shen et al., 2017). As previously noted, bacteriocins have selective antimicrobial activities against ruminal bacteria (both gram-positive and many gram-negative bacteria) because of the disruption of the bacterial cell wall by forming pores and inhibiting important cell wall precursors (Wiedemann et al., 2001) or by inducing oxidative stress in cells (Schaefer et al., 2010). Shen et al. (2017) observed an increased relative abundance of some Gram-positive fibrolytic bacterial genera, such *Pseudobutyrivibrio*, *Butyrivibrio*, *Rikenellaceae*, and *Bacteroidales* that have roles in nutrient digestion (e.g., fiber), with bacteriocin administration. Using pure cultures of rumen bacteria, Demirtaş (2020) observed a stimulated growth of Gram-negative rumen bacteria *M. elsdenii* and *F. succinogenes* as well as some Gram-positive bacteria like *R. albus*, *E. ruminantium*, and *S. bovis*. Such effects may explain the observed improved nutrient digestion with bacteriocins.

#### Ruminal fermentation

Both PNP and CNP lowered ruminal NH_3_–N, with values ranging from 25.2 to 27.7 mg/dL which are greater than the level (8.5 to over 30 mg ammonia-N/dL) for optimum rumen microbial proliferation and activity (Jones and Jones, 2012). As previously noted, bacteriocins decrease ruminal NH_3_–N as a result of suppressing amino acid deamination. However, bacteriocins are proteinous substance that may be susceptible to ruminal proteolysis, previous experiments stated that bacteriocins are binding to ruminal bacteria faster than their degradation (Lee et al., 2002b). Previous experiments (Chen et al., 2017) showed that bacteriocins, such as nisin, decreased the number of hyper ammonia-producing bacteria (*C. aminophilum* and *C. sticklandii*) which may reduce ruminal NH_3_–N production as observed in the present study.

Treatments did not affect ruminal pH, with values (>6) greater than the optimum level (5.6) for ruminal fiber degrading microbial activities and growth (Ryle and Ørskov, 1990). However, both PNP and CNP increased VFA, which may be a result of improved OM, NSC, and fiber digestibility. In the present experiment, increasing the production of propionate and acetate resulted in increased total production of VFA. In their experiment, Shen et al. (2017) reported that nisin greatly increased the relative abundance of *Succinivibrio* and *Selenomonas* which probably contributes to increase propionate via the succinate pathway. They concluded that propionate production through the acrylate pathway was probably weakened by nisin and that the increase in propionate production was via the succinate pathway. Moreover, the accumulation of H_2_ that the methanogens could not utilize was probably rechanneled for propionate formation (Chen et al., 2017).

Almost no experiments have reported increased acetate production with bacteriocins administration to animals; however, many experiments have observed a decrease in acetate production (Shen et al., 2017; Choudhury et al., 2022). On the other hand, the increased acetate production with PNP and CNP may be related to increased fiber digestion (Hernández-González et al., 2021); however, Shen et al. (2017) observed a lowered in vitro acetate production with nisin because of lowered numbers of some Gram-positive fibrolytic bacteria, such as *Ruminococcus* spp., which are major acetate-producing bacteria (Jeyanathan et al., 2014).

#### Blood chemistry measurements

The concentrations of blood total proteins, globulin, glucose, and GPT were within the physiological standards, while the concentrations of albumin, urea-N, and cholesterol were higher than the physiological standards compared to GOT values which were less than the physiological standards for healthy ewes (Jerry Kaneko et al., 2008). This may be related to ewe’s breed (Rahmani sheep). The values of blood measurements were in agreement with those reported for ewes and growing lambs (Anwar et al., 2012; El-Emam et al., 2014). PNP treatment increased blood total proteins and albumin, which are important indicators for improved dietary protein utilization and improved nutritional and physiological status of the ewes as result of the improved nutrient digestibility with PNP. Both PNP and CNP treatments increased serum glucose, which may be associated with the observed enhanced apparent OM and NSC digestibility. Huntington et al. (2006) reported a strong relationship between serum glucose and ruminal propionate because blood glucose is synthesized from ruminal propionate in the liver, and this is corroborated with our data on rumen fermentation

Treatments did not affect the concentrations of serum cholesterol indicating minimal effects on lipid metabolism, and liver function (Žubčić, 2001). Additionally, treatments did not affect the concentrations of serum GOT or GPT, suggesting positive effects of the additives on liver health (Pettersson et al., 2008).

#### Milk yield and composition

Both PNP and CNP treatments increased daily milk production (milk by 10.6% and 8.3%, ECM by 11.3% and 7.3%; FCM by 13.6% and 8.4%, respectively). Improving milk production without affecting intake improved feed efficiency calculated as: milk: intake by 10.3% for both treatments; ECM: intake by 11.8% and 8.8%; FCM: intake by 16.1% and 12.9%, respectively. Improving nutrient digestion and increasing the production of total VFA and propionate are possible reasons for increasing daily milk production. Such results confirm our assumption that additives caused changes in ruminal microbial community composition.

Ruminal propionate concentration is related to increasing the availability of precursors for glucose and lactose synthesis, which positively affect milk yield (Rigout et al., 2003). As previously noted, increasing blood glucose suggests an improved energy status, and can be another reason for increases in milk production in ewes-fed diets supplemented with bacteriocins (Rigout et al., 2003). Moreover, it has been reported that bacteriocins improve gastrointestinal tract health status and reduce pathogens which may reflect in increases in milk production (Alharbi and Alsaloom, 2021; Soltani et al., 2021).

Contents of fat, lactose, and total solids were increased by treatments, which somehow reflected results from nutrient digestibility. It was expected that increasing fiber digestibility may increase fat concentration in milk, while the improved serum glucose was expected to increase lactose concentration. Almost no reports are available about the effect of feeding bacteriocins to lactating animals on milk production and composition; therefore, at this point, the mechanisms behind those results remain unclear.

## CONCLUSIONS

Both bacteriocin-like substance produced from *Lactococcus lactis* ssp. *lactis* or commercial nisin positively affected the performance of ewes at 500 unit/kg DM feed. Bacteriocins improved nutrient digestibility and positively affected ruminal fermentation (increased concentrations of total and individual VFA) and blood chemistry (increased total protein and glucose). Additionally, additives improved milk production and enhanced feed efficiency without affecting milk composition. Therefore, any of the evaluated bacteriocins at 500 unit/kg DM feed/ewe is recommended. Additionally, the like substance bacteriocins produced from *Lactococcus lactis* were superior to the commercial bacteriocin.

## Data Availability

The datasets generated during and/or analyzed during the current study are available from the corresponding author on reasonable request.
